# Effect of Renal Impairment on the Pharmacokinetics and Safety of Doxecitine and Doxribtimine: A Single‐Dose Phase 1 Study

**DOI:** 10.1002/cpdd.70036

**Published:** 2026-02-13

**Authors:** Aravind Mittur, Susan A. VanMeter

**Affiliations:** ^1^ UCB Emeryville CA USA; ^2^ UCB Morrisville NC USA

**Keywords:** deoxycytidine, deoxythymidine, pharmacokinetics, renal impairment, safety, TK2 deficiency

## Abstract

This Phase 1 study investigated the pharmacokinetics and safety of a single dose of the FDA‐approved doxecitine and doxribtimine (266.6 mg/kg; 133.3 mg/kg of deoxycytidine [dC] and deoxythymidine [dT]) in participants with severe (n = 8) or moderate (n = 8) renal impairment (estimated glomerular filtration rate [eGFR] 15–29 mL/min/1.73 m^2^ and 30–59 mL/min/1.73 m^2^, respectively) versus healthy matched controls (eGFR ≥90 mL/min/1.73 m^2^; n = 16 [two groups of eight]). Participants underwent serial sampling to determine total dC and dT plasma concentrations before (baseline) and after dosing (up to 96 h).

Participants with renal impairment had higher baseline‐corrected dC and dT concentrations than controls, which peaked 0.75–1.5 h post‐dose and declined to near baseline levels in ≤18 h. Geometric mean baseline‐corrected plasma maximum concentration and area under the concentration–time curve (respectively) for dC and dT were higher in participants with severe (dC: 7.8 ng/mL and 52.8 h × ng/mL; dT: 18.8 ng/mL and 31.5 h × ng/mL) or moderate (dC: 8.2 ng/mL and 56.4 h × ng/mL; dT: 12.2 ng/mL and 23.7 h × ng/mL) renal impairment than in controls (dC: 4.6–5.3 ng/mL and 25.4–31.8 h × ng/mL; dT: 4.0–7.6 ng/mL and 4.3–12.7 h × ng/mL), with substantial variability. Geometric mean apparent terminal‐phase half‐lives in severe renal impairment, moderate renal impairment, and controls, respectively, were 14.5, 15.3, and 5.2–5.8 h for dC and 3.7, 4.5, and 0.4–1.5 h for dT. One participant experienced treatment‐emergent adverse events (severe renal impairment cohort). In conclusion, renal impairment was associated with increased dC and dT exposure following a single dose of doxecitine and doxribtimine. No safety issues were identified.

Thymidine kinase 2 (TK2) is a mitochondrial matrix enzyme that provides pyrimidine nucleotides for mitochondrial DNA (mtDNA) synthesis and maintenance.^1^, ^2^ It phosphorylates deoxycytidine (dC) and deoxythymidine (dT) to their respective deoxynucleoside monophosphates, which are subsequently phosphorylated to deoxynucleoside triphosphates by other kinases and incorporated into replicating mtDNA.^2^ TK2 deficiency (TK2d) is an mtDNA depletion and/or multiple deletions disease caused by biallelic pathogenic variants of *TK2* in nuclear DNA.^2^, ^3^, ^4^, ^5^ It is an ultra‐rare disease with an estimated prevalence of approximately 1.64 per million people worldwide,^6^ with an age of TK2d symptom onset ≤12 years in >80% of cases.^4^

As a consequence of the proximal myopathy caused by TK2d, which can present as progressive weakness of limb, neck, facial, oropharyngeal, and respiratory muscles,^2^ many people with TK2d lose the ability to walk, eat, and breathe independently.^3^, ^4^, ^7^, ^8^, ^9^ TK2d increases the risk of early death, primarily due to respiratory failure.^4^, ^5^, ^8^, ^9^ Although occurring along a continuous clinical spectrum, it has been suggested that stratifying by age of TK2d symptom onset may be useful when describing clinical forms of the disease.^2^, ^4^ Age of symptom onset ≤2 years is associated with rapid progression to early death; age of symptom onset >2 to ≤12 years is associated with a slower progression to early death and, in most cases, to loss of ambulation within 10 years of disease onset and progression to use of ventilatory support; and age of symptom onset >12 years is associated with subclinical or mild myopathy at disease onset in adolescence through adulthood, slower progression with retained ability to walk, and frequent use of ventilatory support.^2^, ^4^, ^10^ However, natural history data in the last group are limited, making the disease trajectory and prognosis unclear.

As of November 2025, doxecitine and doxribtimine, a 1:1 mixture of equal weights of dC and dT, represents the first FDA‐approved treatment for TK2d for patients with an age of symptom onset ≤12 years.^11^, ^12^ Previously, treatment was reliant on supportive approaches to managing symptoms and preventing complications. Doxecitine and doxribtimine targets the underlying pathology of TK2d by increasing levels of substrate available for residual mitochondrial TK2 activity and for utilization by thymidine kinase 1 and deoxycytidine kinase in the cytosol.^13^ In preclinical testing, doxecitine and doxribtimine delayed disease onset and prolonged the lifespan of TK2‐deficient mice, restoring mtDNA copy number, as well as respiratory chain enzyme activities and levels.^13^, ^14^ Although mouse models indicate that only dT corrects the TK2d phenotype, both doxecitine and doxribtimine are needed in equal amounts to balance the deoxynucleotide triphosphate pools and prevent inhibition of DNA synthesis by excess thymidine.^15^, ^16^, ^17^ An analysis of pooled data from patients treated with pyrimidine nucleos(t)ides in retrospective (NCT03701568 and NCT05017818) and prospective (NCT03845712 [data cut‐off date: March 15, 2024]) studies, and company‐supported expanded access programs indicated that treatment improved survival and functional outcomes in patients with TK2d, particularly in those with age of symptom onset ≤12 years, and had an acceptable safety profile.^18^, ^19^, ^20^, ^21^

dC and dT are primarily catabolized by cytidine deaminase and thymidine phosphorylase, respectively, to the nucleobases and the 2‐deoxy‐α‐D‐ribose 1‐phosphate moiety.^22^ Intermediate metabolites of dC catabolism are deoxyuridine, uracil, and dihydrouracil, with end products β‐alanine, ammonia, and carbon dioxide (CO_2_). Thymine, the pyrimidine nucleobase in dT, is subsequently metabolized to dihydrothymine and ultimately to γ‐amino‐isobutyric acid and CO_2_. dC and dT are not subject to cytochrome P450 (CYP)‐mediated metabolism.^23^ Non‐clinical and clinical studies have suggested that dC and dT and their metabolites are eliminated by renal excretion with extensive hepatic/extrahepatic metabolism characterized by high hepatic extraction.^24^ The human liver expresses the full complement of catabolic enzymes for the two pyrimidine nucleosides (dC and dT).^25^ Pharmacokinetic (PK) analyses of doxecitine and doxribtimine in healthy adults have been published previously.^26^ In a PK analysis of two Phase 1 studies of doxecitine and doxribtimine, peak plasma concentration and extent of exposure to dC and dT were shown to increase with dose over endogenous (pre‐dose) concentrations, confirming the oral bioavailability of the treatment.^26^ Administration with a high‐fat, high‐calorie meal was also shown to significantly increase their exposure.^26^ Median time to maximum exposure under fasting conditions ranged from 1 to 2 h (delayed to 2–4 h after administration with food), and post‐dose plasma dC and dT concentrations returned to nearly baseline concentrations after 8–12 h.^26^ Renal clearance played a minor role in the elimination of unchanged (intact) dC and dT in the intended clinical dose range.^26^ However, it is of interest to determine whether renal impairment adversely affects the overall clearance of dC and dT, particularly during long‐term administration in the setting of TK2d therapy. As such, this study was conducted to assess the impact of severe and moderate renal impairment on the PK profile of and systemic exposure to dC and dT following administration of a single dose of doxecitine and doxribtimine.

The primary objective of the study was to evaluate the PK of doxecitine and doxribtimine in adult participants with severe renal impairment or moderate renal impairment compared with those with normal renal function (healthy matched controls). The secondary objective was to evaluate the safety and tolerability of doxecitine and doxribtimine in all study participants.

## Methods

### Study Design

The investigators of this study obtained Institutional Review Board approval for the protocol and the written informed consent from the IntegReview Institutional Review Board (Austin, TX, USA) and the Advarra Institutional Review Board (Columbia, MD, USA) prior to study initiation at the participating centers (Alliance for Multispecialty Research, LLC, Knoxville, TN, USA; Orlando Clinical Research Center, Orlando, FL, USA; Advanced Pharma CR, LLC, Miami, FL, USA). This study was conducted in conformance with the provisions of the Declaration of Helsinki and all revisions thereof, and in accordance with the US FDA Code of Federal Regulations and the International Council for Harmonisation of Technical Requirements for Registration of Pharmaceuticals for Human Use E6 guidelines on Good Clinical Practice. Written informed consent was obtained from each participant at the screening visit. The study was designed in accordance with FDA guidance on PK studies in participants with impaired renal function.^27^

This was a Phase 1, open‐label, parallel‐group study comprising a screening period, a baseline visit (Day −1), and study drug treatment (Day 1), followed by a confinement period up to Day 5 and a follow‐up telephone call on Day 14 (±2 days). The study was conducted in two parts in three clinical centers in the USA between January 25, 2021, and May 11, 2021. Part 1 compared adult participants with severe renal impairment (estimated glomerular filtration rate [eGFR] 15–29 mL/min/1.73 m^2^) with matched controls who had normal renal function (eGFR ≥90 mL/min/1.73 m^2^), and Part 2 evaluated adult participants with moderate renal impairment (eGFR 30–59 mL/min/1.73 m^2^) versus matched controls with normal renal function (eGFR ≥90 mL/min/1.73 m^2^). Part 1 and Part 2 were conducted in conjunction, as permitted by participant availability and investigator resources. Renal function was estimated using the Cockcroft–Gault or Modification of Diet in Renal Disease equation for eGFR. The control participants were matched to one participant with severe renal impairment and one participant with moderate renal impairment, if possible, and matched to the corresponding renal impairment group with respect to age ±10%, sex, and body mass index (BMI) ±15%. The matched controls received treatment once a matched participant with renal impairment had completed the study.

### Key Inclusion and Exclusion Criteria

All participants eligible for inclusion in the study were men or women (aged ≥18 years at screening) with a BMI of ≥18.5 kg/m^2^ at screening who were non‐smokers, ex‐smokers, or light smokers and had vital signs within designated ranges, or without clinically significant deviations outside of the ranges likely to impact study conduct (i.e., after approximately 5 min resting in seated position, a systolic blood pressure of 90–175 mmHg, diastolic blood pressure of 50–100 mmHg, and heart rate of >45–100 beats per minute). Also, participants were of non‐childbearing potential, surgically sterile, or postmenopausal (women), or using a highly effective method of contraception (for sexually active men and their partners of childbearing potential). For participants with renal impairment, a stable dose of medication or treatment regimen for ≥2 weeks prior to study dosing was necessary, while matched controls had to be medically healthy and without clinically significant deviations of clinical laboratory test results from the normal reference range likely to impact study conduct.

Key exclusion criteria were: presence of clinically significant physical, laboratory, or electrocardiogram (ECG) findings with potential to interfere with any aspect of the study conduct or interpretation of results (participants considered not eligible because of ECG findings may have been included at the discretion of the investigator and medical monitor); history of any clinically relevant psychiatric, pulmonology, hepatic, pancreatic, endocrine, cardiovascular, neurological, hematological, or gastrointestinal abnormality; unstable glucose control with glycated hemoglobin >9.5% at screening; history of bariatric surgery or any other gastrointestinal surgery that may have induced malabsorption or history of any major surgery or trauma in the 6 months prior to Day 1, or surgery planned during the study; history of active infection in the 14 days prior to Day 1 if deemed clinically significant; existence of any ongoing medical condition, signs, or symptoms considered to potentially interfere with the PK of the study treatment or safety or tolerability measurements; and history of hepatic disease or significantly abnormal liver function tests (alanine transaminase, aspartate transaminase, and total bilirubin >2 times the upper limit of normal at screening), or of renal disease (participants considered not eligible because of laboratory results may have had tests repeated once during screening at the discretion of the investigator to determine eligibility).

### Participant Disposition and Characteristics

In total, 32 participants were enrolled and included in all three analysis populations (i.e., safety, PK, and PK analysis of variance [ANOVA]; Table 1). No participants discontinued treatment, with all groups completing the study.

**Table 1:** Demographic and Baseline Characteristics (Safety Population)

	Part 1			Part 2			
	Severe Renal Impairment (n = 8)	Normal Renal Function (n = 8)	Total (N = 16)	Moderate Renal Impairment (n = 8)	Normal Renal Function (n = 8)	Total (N = 16)	Overall (N = 32)
Age (years)							
Mean (SD)	65.1 (10.8)	59.8 (7.9)	62.4 (9.6)	65.4 (9.1)	65.3 (10.0)	65.3 (9.2)	63.9 (9.4)
Median (min, max)	60.0 (54, 81)	59.0 (51, 76)	60.0 (51, 81)	66.0 (50, 76)	64.5 (50, 80)	65.5 (50, 80)	62.0 (50, 81)
Sex							
Female, n (%)	3 (37.5)	3 (37.5)	6 (37.5)	3 (37.5)	3 (37.5)	6 (37.5)	12 (37.5)
Male, n (%)	5 (62.5)	5 (62.5)	10 (62.5)	5 (62.5)	5 (62.5)	10 (62.5)	20 (62.5)
Race							
Black/African American, n (%)	1 (12.5)	1 (12.5)	2 (12.5)	1 (12.5)	3 (37.5)	4 (25.0)	6 (18.8)
White, n (%)	7 (87.5)	7 (87.5)	14 (87.5)	7 (87.5)	5 (62.5)	12 (75.0)	26 (81.3)
Ethnicity							
Hispanic/Latino, n (%)	7 (87.5)	8 (100.0)	15 (93.8)	8 (100.0)	8 (100.0)	16 (100.0)	31 (96.9)
Not Hispanic/Latino, n (%)	1 (12.5)	0 (0)	1 (6.3)	0 (0)	0 (0)	0 (0)	1 (3.1)
Weight (kg)							
Mean (SD)	69.6 (15.8)	78.6 (13.6)	74.1 (15.0)	79.2 (11.2)	84.7 (13.4)	81.9 (12.3)	78.0 (14.1)
Median (min, max)	69.0 (45.9, 86.7)	79.8 (55.7, 100.0)	76.2 (45.9, 100.0)	81.8 (55.8, 89.7)	83.5 (68.6, 103.0)	82.5 (55.8, 103.0)	79.5 (45.9, 103.0)
Baseline BMI (kg/m^2^)							
Mean (SD)	27.8 (4.7)	28.1 (3.3)	28.0 (3.9)	29.6 (3.2)	28.7 (1.8)	29.1 (2.6)	28.55 (3.3)
Median (min, max)	28.7 (19.1, 33.9)	28.7 (21.8, 31.2)	28.7 (19.1, 33.9)	30.1 (25.6, 35.7)	28.4 (26.8, 31.7)	29.2 (25.6, 35.7)	29.1 (19.1, 35.7)
eGFR (mL/min/1.73 m^2^)							
Mean (SD)	23.1 (7.4)^a^	101 (11.8)		44.7 (8.0)	93.3 (4.3)		
Median (min, max)	27.2 (15, 29)^a^	97.2 (91.0, 124)		45.6 (31.3, 55.0)	91.7 (90.7, 104)		
SBP (mmHg)							
Mean (SD)	132.9 (10.1)	124.4 (10.2)	128.6 (10.7)	131.0 (9.2)	122.1 (17.3)	126.6 (14.1)	
Median (min, max)	133.0 (119, 148)	129.5 (110, 135)	131.0 (110, 148)	134.0 (105, 149)	115.0 (105, 149)	130.5 (105, 149)	
DBP (mmHg)							
Mean (SD)	72.6 (9.0)	77.4 (8.3)	75.0 (8.7)	75.3 (7.6)	77.8 (4.2)	76.5 (6.1)	
Median (min, max)	75.5 (53, 83)	76.5 (69, 95)	76.0 (53, 95)	78.5 (63, 83)	77.0 (73, 86)	77.5 (63, 86)	
Heart rate (beats/minute)							
Mean (SD)	66.8 (7.3)	72.6 (9.2)	69.7 (8.6)	74.4 (6.7)	67.5 (7.3)	70.9 (7.7)	
Median (min, max)	64.0 (58, 80)	75.0 (60, 83)	68.0 (58, 83)	77.0 (62, 81)	70.0 (51, 73)	72.5 (51, 81)	
Pre‐dose dC plasma concentration (ng/mL)							
Mean (SD)	3.0 (0.7)	3.0 (0.7)		2.6 (0.8)	3.0 (1.1)		
Median (min, max)	2.8 (2.1, 3.7)	2.8 (1.8, 4.1)		2.5 (1.7, 4.2)	2.9 (1.1, 4.5)		
Pre‐dose dT plasma concentration (ng/mL)							
Mean (SD)	0.2 (0.3)	0.2 (0.3)		0.1 (0.2)	0.1 (0.2)		
Median (min, max)	0.0 (0.0, 0.9)	0.0 (0.0, 0.7)		0.0 (0.0, 0.6)	0.0 (0.0, 0.7)		

a n = 7; for one participant in this group, Cockcroft–Gault method was used to ensure eligibility criteria were met, and the eGFR value was not included in the summary.

BMI, body mass index; DBP, diastolic blood pressure; dC, deoxycytidine; dT, deoxythymidine; eGFR, estimated glomerular filtration rate; max, maximum; min, minimum; SBP, systolic blood pressure; SD, standard deviation.

Participant demographic and baseline characteristics are summarized in Table 1. Except for eGFR, baseline characteristics were similar between participants with severe and moderate renal impairment and their matched controls. No participants were on dialysis at the point of study initiation, and no participants required dialysis during the study.

### Treatment

The study treatment was doxecitine and doxribtimine at a 1:1 ratio by weight, as a powder for reconstitution (in apple juice or water) as an oral solution. The study drug was supplied as a single sachet containing 2.0 g of dC and 2.0 g of dT. This configuration provided dC and dT components to achieve a nominal dosing solution concentration after reconstitution of 50 mg/mL of both dC and dT. All participants received a single oral dose of doxecitine and doxribtimine (266.6 mg/kg, containing 133.3 mg/kg of each component) on Day 1 (the dose is the highest strength, approximately equivalent to one of three daily doses at the intended maintenance dosage of 800 mg/kg/day). The treatment was administered with approximately 240 mL of nonrefrigerated, noncarbonated water following an overnight fast of ≥10 h. No food was permitted for ≥4 h after dosing on Day 1. Water was permitted as desired, except for ≤1 h before or after dosing. For participants with renal impairment, any required concomitant medications were administered ≥4 h before study treatment dosing.

### Assessments

PK parameters were calculated for baseline‐corrected and baseline‐uncorrected (actual) dC and dT concentration data using noncompartmental methods. Plasma and urine concentration data were analyzed and presented separately for dC and dT. Plasma samples were collected before dosing and at 0.5, 1, 2, 3, 4, 6, 8, 10, 12, 18, 24, 48, 72, and 96 h after dosing to determine maximum observed concentration (C_max_); time to reach C_max_ (t_max_); area under the concentration–time curve (AUC) from time 0 to time of the last quantifiable concentration (AUC_0–t_); AUC from time 0 to 4 h (AUC_0–4_); AUC from time 0 to 6 h (AUC_0–6_); AUC from time 0 to 8 h (AUC_0–8_); AUC from time 0 to 96 h (AUC_0–96_); AUC from time 0 extrapolated to infinity (AUC_0–inf_); percentage of AUC_0–inf_ extrapolated (AUC_%extrap_); last measurable plasma concentration (C_last_); time that C_last_ was observed (T_last_); apparent terminal‐phase half‐life (t_½_); rate constant associated with the terminal (log‐linear) phase of the concentration–time curve (λ_z_); apparent oral clearance (CL/F); and apparent volume of distribution during the terminal phase (V_z_/F).

dC and dT were extracted from plasma samples by a single solid‐phase extraction procedure and quantified by liquid chromatography electrospray ionization tandem mass spectrometry (LC/ESI/MS/MS) in positive ion mode using [^15^N_3_]‐deoxycytidine and [D_3_]‐deoxythymidine as internal standards for dC and dT, respectively. The validated range of dC and dT concentration in human plasma was 0.500–200 ng/mL. Stability of dC and dT in human plasma was demonstrated for: 106 h at 5°C; ≥798 days at −20°C and −80°C, using eight cycles of freeze/thaw (−80°C to ice bath); and 26 h at ambient temperature.

Urine samples were collected ≤2 h before dosing (voided) and then quantitatively collected and pooled during 0–4, 4–8, 8–12, 12–24, 24–48, 48–72, and 72–96 h following dosing to determine the amount of unchanged dC and dT excreted in the urine through time t (Ae), amount of unchanged dC and dT excreted in the urine collection interval from t' to t″ (Ae_(t'–t″)_), renal clearance (CL_r_), fraction of dC and dT excreted in urine (Fe), and fraction of the unchanged dC and dT excreted in urine over the interval from time 0 to 8 h (Fe_0–8_). Urine concentrations of dC and dT were quantified by a validated method using LC/ESI/MS/MS in positive ion mode using [^15^N_3_]‐deoxycytidine and [D_3_]‐deoxythymidine as internal standards for dC and dT, respectively. The procedure used one extraction by separate high‐performance liquid chromatography methods for each analyte. The method was validated to quantify dC and dT between 1.00 and 1000 ng/mL and between 5.00 and 1000 ng/mL, respectively, in human urine. Stability of dC and dT in urine was demonstrated for: ≥175 h at 5°C; ≥9 days at −80°C, using ≥4 cycles of freeze/thaw (−80°C to ice bath); and ≥274 h at ambient temperature. The methods for the determination of dC and dT in human plasma and urine were precise, accurate, sensitive, and selective over the concentration range and were considered suitable for the analyses of PK studies.

Safety and tolerability assessments included physical examinations, 12‐lead ECGs, vital signs, clinical laboratory evaluations (hematology, clinical chemistry, and urinalysis), and adverse event (AE) and serious AE monitoring.

### Statistical Analysis

No formal sample size calculation was performed. The number of participants per renal function group was selected for practical reasons. Analysis populations included: the safety population, defined as all participants who received the dose of study treatment; the PK population, defined as all participants who had sufficient concentration data to allow estimation of ≥1 PK parameter; and the PK ANOVA population, defined as all participants with severe or moderate renal impairment matched with a single healthy control participant (who was dosed), who had sufficient PK samples collected such that the two key PK parameters (C_max_ and AUC_0–t_) were generated for both participants and could be included in a formal ANOVA statistical analysis.

Descriptive statistics (number [n], mean, coefficient of variation [CV%], standard deviation [SD], median, minimum, maximum, geometric mean [GM], and geometric CV%) were calculated for baseline‐corrected and baseline‐uncorrected dC and dT plasma concentrations, and for dC and dT urine amounts following a single dose of study treatment for participants with severe or moderate renal impairment and their corresponding matched controls (PK population). Urine excretion data were summarized for all time intervals by renal function group. Regression analysis was used to assess the relationship between log‐transformed baseline‐corrected PK parameters and eGFR. Comparisons between renal function groups were evaluated by an analysis of the log‐transformed primary baseline‐corrected and baseline‐uncorrected PK parameters (C_max_ and AUC_0–t_), with ANOVA for dC and dT performed separately. The ANOVA was performed on log‐transformed C_max_ and AUC_0–t_ for dC and dT for the severe and moderate renal impairment groups and the corresponding matched control groups by including renal function group as a fixed effect and participant as a random effect (PK ANOVA population). Safety data (treatment‐emergent AEs [TEAEs], clinical laboratory data, vital signs, ECG parameters) were analyzed descriptively in the safety population.

## Results

### PK Evaluation—dC

**Figure 1:**
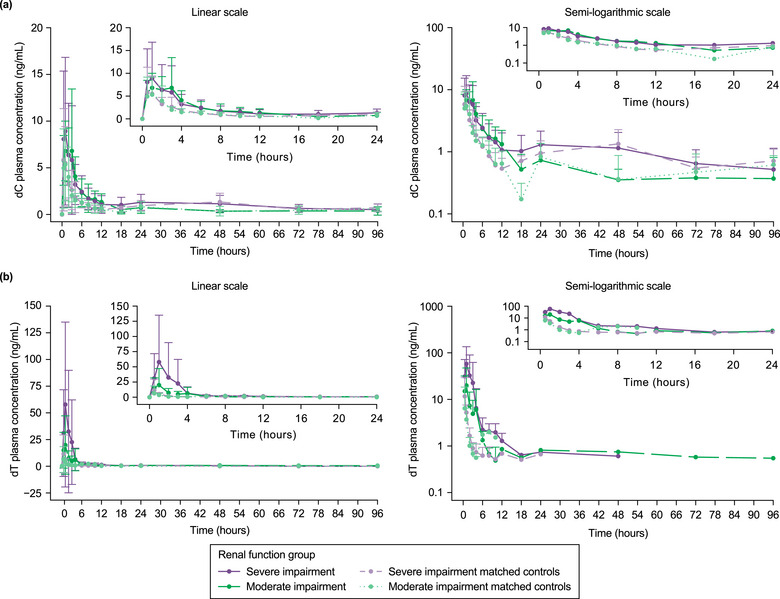
Baseline‐corrected plasma concentration–time profiles for (a) dC and (b) dT following a single dose of study treatment (PK population). Data shown are mean values with SD as error bars. Both linear (left) and semi‐logarithmic (right) scales are presented to illustrate concentration changes over time. The lower limit of quantification for both analytes in plasma was 0.5 ng/mL. Part 1 of the study compared participants with severe renal impairment and matched controls. Part 2 of the study compared participants with moderate renal impairment and matched controls. dC, deoxycytidine; dT, deoxythymidine; PK, pharmacokinetic; SD, standard deviation.

At baseline, dC plasma concentrations were similar across all renal function groups. Mean pre‐dose dC concentrations ranged from 2.6 ng/mL in participants with moderate renal impairment to 3.0 ng/mL for all other participants, including those with severe renal impairment and all matched controls. Baseline‐corrected post‐dose dC plasma concentrations were higher in participants with renal impairment than in those with normal renal function. Plasma dC concentrations peaked at approximately 0.75–1.5 h after dosing and declined to baseline levels at approximately 12–18 h in all groups (Figure 1a). Baseline‐uncorrected plasma dC concentrations are shown in Figure S1a.

**Figure 2:**
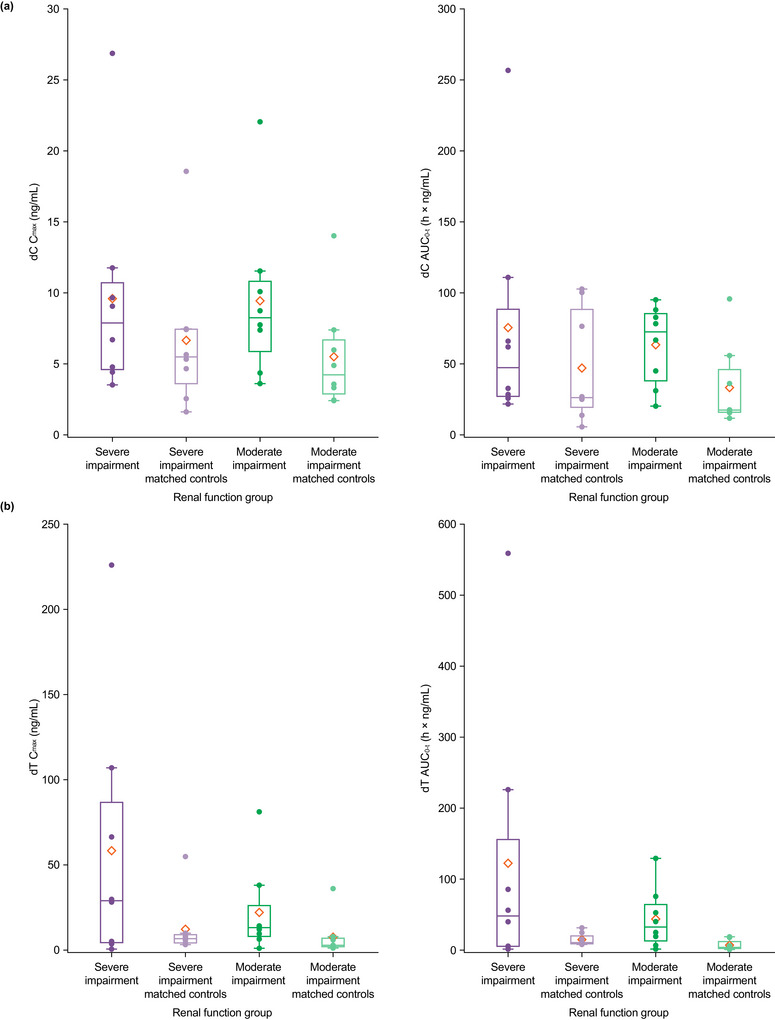
Baseline‐corrected plasma C_max_ and AUC_0–t_ for (a) dC and (b) dT by renal function group (PK population). Horizontal lines in the boxes show the median, and the top and bottom edges of the boxes show Q3 and Q1. Diamond symbols show the mean. Lower and upper whiskers in the box plot are the minimum and maximum observed values within the range [Q1 − 1.5 × IQR] and [Q3 + 1.5 × IQR], where IQR is the interquartile range (Q3–Q1). Observed values outside this range are marked as outliers (circles above and below the whiskers). AUC_0–t_, area under the concentration–time curve from time 0 to time of the last quantifiable concentration; C_max_, maximum observed plasma concentration; dC, deoxycytidine; dT, deoxythymidine; IQR, interquartile range; PK, pharmacokinetic; Q, quartile.

Plasma exposure of dC was increased in participants with renal insufficiency relative to matched controls (Table 2; Figure 2a; Figure S2a). GM C_max_ and AUC_0–t_ values of dC were higher in the renal impairment groups than in matched controls. Inter‐participant variability (geometric CV%) for baseline‐corrected dC C_max_ and AUC_0–t_ values ranged from 61% to 135% in all renal function groups, including matched controls (Table 3). Compared with matched controls, baseline‐corrected plasma dC C_max_ and AUC_0–t_ were 48% and 66% higher, respectively, in participants with severe renal impairment, and 77% and 122% higher, respectively, in participants with moderate renal impairment.

**Table 2:** Effect of Renal Impairment on Baseline‐Corrected C_max_ and AUC_0–t_ (PK ANOVA Population)

PK Parameter	Renal Function Status	n	Mean (SD)	Geometric LSM	Ratio of Geometric LSM (T/R)	90% CI for Geometric LSM Ratio (T/R)
dC						
C_max_ (ng/mL)	Moderate impairment	8	9.44 (5.8)	8.2	1.768	1.058, 2.953
	Matched control	8	5.50 (3.9)	4.6		
	Severe impairment	8	9.60 (7.6)	7.8	1.481	0.804, 2.729
	Matched control	8	6.66 (5.2)	5.3		
AUC_0–t_ (h × ng/mL)	Moderate impairment	8	63.4 (27.9)	56.4	2.219	1.244, 3.956
	Matched control	8	33.3 (29.3)	25.4		
	Severe impairment	8	75.5 (79.1)	52.8	1.659	0.726, 3.791
	Matched control	8	47.0 (39.6)	31.8		
dT						
C_max_ (ng/mL)	Moderate impairment	8	22.1 (26.2)	12.2	3.099	1.087, 8.836
	Matched control	8	7.6 (11.7)	4.0		
	Severe impairment	8	58.4 (76.9)	18.8	2.462	0.639, 9.477
	Matched control	8	12.3 (17.4)	7.6		
AUC_0–t_ (h × ng/mL)	Moderate impairment	8	43.8 (42.3)	23.7	5.468	1.792, 16.684
	Matched control	8	7.0 (7.18)	4.3		
	Severe impairment	8	122 (191)	31.5	2.479	0.663, 9.274
	Matched control	8	14.5 (8.7)	12.7		

The lower limit of quantification for both analytes in plasma was 0.500 ng/mL.

ANOVA, analysis of variance; AUC_0–t_, area under the concentration–time curve from time 0 to time of last quantifiable concentration; CI, confidence interval; C_max_, maximum observed concentration; dC, deoxycytidine; dT, deoxythymidine; LSM, least‐squares mean; PK, pharmacokinetics; R, reference (matched normal); SD, standard deviation; T, test (severe or moderate impairment).

**Table 3:** Summary of Baseline‐Corrected dC and dT PK Parameters Following Single‐Dose Administration of Study Treatment (PK Population)

		Part 1				Part 2			
		dC		dT		dC		dT	
PK Parameter	Statistic	Severe Renal Impairment (n = 8)	Normal Renal Function (n = 8)	Severe Renal Impairment (n = 8)	Normal Renal Function (n = 8)	Moderate Renal Impairment (n = 8)	Normal Renal Function (n = 8)	Moderate Renal Impairment (n = 8)	Normal Renal Function (n = 8)
C_max_ (ng/mL)	n	8	8	8	8	8	8	8	8
	GM	7.8	5.3	18.8	7.6	8.2	4.6	12.2	4.0
	GeoCV%	73.0	84.3	697	109	61.2	65.9	201	154
	Mean (SD)	9.6 (7.5)	6.7 (5.2)	58.4 (76.9)	12.3 (17.4)	9.4 (5.7)	5.5 (3.9)	22.1 (26.2)	7.6 (11.7)
T_max_ (h)	n	8	8	8	8	8	8	8	8
	Median	0.8	0.8	1.0	0.5	1.5	0.8	1.0	0.8
	(min, max)	(0.5, 2.0)	(0.5, 3.0)	(0.5, 2.0)	(0.5, 1.0)	(0.5, 3.0)	(0.5, 3.0)	(0.5, 3.0)	(0.5, 1.0)
AUC_0–t_ (h × ng/mL)	n	8	8	8	8	8	8	8	8
	GM	52.8	31.8	31.5	12.7	56.4	25.4	23.7	4.3
	GeoCV%	103	135	817	56.1	61.0	85.3	261	147
	Mean (SD)	75.5 (79.1)	47.0 (39.6)	122 (191)	14.5 (8.7)	63.4 (27.9)	33.3 (29.3)	43.8 (42.3)	7.0 (7.2)
AUC_0–6_ (h × ng/mL)	n	8	8	4	6	8	8	5	1
	GM	24.7	14.7	118	9.4	26.3	14.3	37.6	11.7
	GeoCV%	63.0	56.1	193	29.3	49.7	41.4	104	NC
	Mean (SD)	29.7 (24.6)	16.4 (7.5)	204 (223)	9.7 (3.0)	28.8 (12.4)	15.5 (8.10)	50.6 (45.4)	11.7 (NC)
AUC_0–inf_ (h × ng/mL)	n	4	2	3	3	2	2	3	1
	GM	55.1	52.8	97.2	14.6	53.9	30.4	52.4	3.0
	GeoCV%	65.1	131	111	85.5	83.5	110	117	NC
	Mean (SD)	63.0 (37.4)	66.6 (57.3)	126 (109)	17.7 (13.7)	61.2 (40.9)	36.6 (28.9)	68.2 (55.9)	3.0 (NC)
t_½_ (h)	n	6	2	3	3	2	3	4	1
	GM	14.5	5.8	3.7	1.5	15.3	5.2	4.5	0.4
	GeoCV%	129	192	434	84.9	71.7	126	1100	NC
	Mean (SD)	20.6 (17.2)	8.1 (8.1)	9.6 (14.2)	1.8 (1.2)	16.9 (10.2)	7.0 (6.5)	29.9 (56.6)	0.4 (NC)
V_z_/F (L)	n	4	2	3	3	2	2	3	1
	GM	2,100,000	1,720,000	524,000	1,490,000	4,850,000	3,450,000	444,000	2,380,000
	GeoCV%	91.3	24.1	193	67.9	9.7	46.4	63.7	NC
	Mean (SD)	2,640,000 (2,060,000)	1,740,000 (410,000)	820,000 (828,000)	1,710,000 (1,120,000)	4,860,000 (468,000)	3,620,000 (1,550,000)	492,000 (239,000)	2,380,000 NC
CL/F (L/h)	n	4	2	3	3	2	2	3	1
	GM	158,000	206,000	99,400	673,000	220,000	439,000	204,000	3,830,000
	GeoCV%	79.5	132	88.8	97.2	85.5	117	108	NC
	Mean (SD)	185,000 (98,500)	261,000 (226,000)	120,000 (85,200)	810,000 (499,000)	251,000 (170,000)	537,000 (438,000)	263,000 (224,000)	3,830,000 (NC)
Ae (ng)	n	8	8	8	7	8	8	8	8
	GM	4418	11,429	6865	3362	18,110	27,138	26,631	8420
	GeoCV%	243	262	210	11.2	560	201	384	729
	Mean (SD)	8041.8 (10,432.7)	17,795.1 (20,189.4)	7980.8 (12,427.6)	963.6 (1652.8)	32,957.0 (20,108.2)	39,976.3 (65,134.8)	63,207.9 (72,068.0)	24,251.8 (56,382.8)
CL_r_ (L/h)	n	8	8	8	7	8	8	8	8
	GM	0.01	0.04	0.10	0.29	0.06	0.08	1.10	0.66
	GeoCV%	246	250	157	55.2	429	177	1862	1856
	Mean (SD)	0.02 (0.03)	0.05 (0.06)	0.1 (0.2)	0.09 (0.2)	0.1 (0.06)	0.1 (0.2)	18.4 (47.8)	1.8 (3.3)

The lower limit of quantification for both analytes in plasma was 0.500 ng/mL; the lower limits of quantification for dC and dT in urine were 1.00 ng/mL and 5.00 ng/mL, respectively.

Ae, amount excreted in urine; AUC, area under the concentration–time curve; AUC_0–6_, AUC from time 0 to 6 h; AUC_0–inf_, AUC from time 0 extrapolated to infinity; AUC_0–t_, AUC from time 0 to time of last quantifiable concentration; CL/F, apparent oral clearance; CL_r_, renal clearance; C_max_, maximum observed concentration; dC, deoxycytidine; dT, deoxythymidine; GeoCV%, geometric percent coefficient of variation; GM, geometric mean; max, maximum; min, minimum; NC, not calculable; PK, pharmacokinetics; SD, standard deviation; t_½_, apparent terminal‐phase half‐life; T_max_, time to C_max_; V_z_/F, apparent volume of distribution during the terminal phase.

For the dC PK parameters estimated for baseline‐uncorrected plasma concentrations, ratios of geometric least‐squares mean C_max_ and AUC_0–t_ in the treatment groups were close to 1.0, but the respective 90% confidence intervals were outside the 80%–120% range (Table S1).

Estimates of t_½_ of dC tended to be longer in participants with severe and moderate renal impairment than in matched controls (14.5 [severe] and 15.3 [moderate] h vs 5.8 and 5.2 h, respectively), although data were only evaluable in a limited number of participants owing to the adjusted R^2^ < .8 (Table 3).

Negligible amounts of intact dC were excreted in urine. Renal clearance (GM) of dC tended to be lower in participants with severe renal impairment (0.01 L/h) than in matched controls (0.04 L/h), and slightly lower in participants with moderate renal impairment (0.06 L/h) than in matched controls (0.08 L/h; Table 3).

### PK Evaluation—dT

Baseline dT plasma concentrations were similar across all renal function groups, with mean pre‐dose values ranging from 0.1 ng/mL in participants with moderate renal impairment and their matched controls to 0.2 ng/mL in those with severe renal impairment and their matched controls. Baseline‐corrected post‐dose dT plasma concentrations were higher in participants with renal impairment than in those with normal renal function. Plasma dT concentrations peaked at approximately 0.75–1.0 h after dosing and declined to baseline levels after approximately 4–6 h (Figure 1b). Baseline‐uncorrected plasma dT concentrations are shown in Figure S1b.

Plasma exposure of dT was increased in participants with renal insufficiency relative to matched controls (Table 2; Figure 2b; Figure S2b). GM C_max_ and AUC_0–t_ values of dT were higher in the renal impairment groups than in matched controls. For baseline‐corrected dT C_max_ and AUC_0–t_, inter‐patient variability ranged from 56.1% to 261% in matched controls and the moderate renal impairment group, and from 697% to 817% in the severe renal impairment group (Table 3). Baseline‐corrected plasma dT C_max_ and AUC_0–t_ were 146% and 148% higher, respectively, in participants with severe renal impairment than in matched controls, and 210% and 447% higher, respectively, in participants with moderate renal impairment than in matched controls.

Increases in C_max_ and AUC_0–t_ were evident for baseline‐uncorrected dT plasma PK parameters in participants with impaired renal function compared with matched controls, albeit of smaller magnitudes than for baseline‐corrected exposure (Table S1).

Estimates of dT t_½_ tended to be longer in participants with severe (3.7 h) and moderate (4.5 h) renal impairment than in matched controls (1.5 and 0.4 h, respectively; Table 3).

Negligible amounts of intact dT were excreted in urine. For the urine dT data, parameters should be considered an estimate because the incurred sample reanalysis results did not meet the acceptance criterion of two‐thirds of the reanalyzed concentrations within ±20% of the reference (initial) measured concentration values. Renal clearance (GM) of dT tended to be lower in participants with severe renal impairment (0.10 L/h) than in matched controls (0.29 L/h), and higher in participants with moderate renal impairment (1.10 L/h) than in matched controls (0.66 L/h; Table 3).

### Effects of eGFR on Plasma dC and dT Exposure

Exploratory regression analysis of PK parameters against eGFR showed an increase in baseline‐adjusted plasma dC and dT exposure (in terms of baseline‐adjusted C_max_ and AUC_0–t_) with decreased renal function. The negative relationship was statistically significant (*P* < .05) but weak (coefficient of determination [R^2^] < .274) (Figure S3).

### Safety Evaluation

Of the 32 participants who received treatment, one participant (3.1%) experienced TEAEs; this participant had severe renal impairment. The TEAEs reported for this participant were anemia, electrolyte imbalance, hyperkalemia, and renal impairment (all were Grade 2 in severity, started on Day 15, were ongoing, and were considered possibly related to the study drug). Clinically significant laboratory abnormalities were recorded for this participant and were related to all TEAEs reported (anemia, electrolyte imbalance, hyperkalemia, and renal impairment). No other participants had clinically significant laboratory abnormalities. There were no serious TEAEs, no TEAEs of Grade 3 severity or above, and no TEAEs that led to death. There were also no clinically significant vital sign measurements or ECG findings, and no clinically meaningful trends identified in observed values of ECG parameters or changes from baseline.

## Discussion

This was a Phase 1, open‐label, parallel‐group study that evaluated PK parameters, safety, and tolerability following a single therapeutically relevant dose of doxecitine and doxribtimine in adult participants with severe or moderate renal impairment compared with matched control participants with normal renal function. A single oral dose of 266.6 mg/kg (133.3 mg/kg of dC and 133.3 mg/kg of dT) was used in this study, which is equivalent to the highest daily dosage under clinical investigation (800 mg/kg/day [400 mg/kg/day of dC and 400 mg/kg/day of dT]; based on a three times daily dosing regimen). This dose had previously been well tolerated by healthy adult participants.^26^

Analysis of plasma concentrations revealed that dC and dT were rapidly absorbed following administration of a single oral dose of doxecitine and doxribtimine in all renal function groups, with concentrations of both drug substances returning to near baseline (pre‐dose) values within approximately 18 h. Baseline‐corrected plasma dC and dT exposures (based on C_max_ and AUC_0–t_) were characterized by substantial variability; exposures were similar for participants with severe and moderate renal impairment and were higher for these participants than for healthy matched controls. The observation that baseline (pre‐dose) plasma concentrations of intact dC and dT were similar in the renally impaired groups and healthy matched controls suggests that their endogenous plasma concentrations are not intrinsically affected by the degree of renal impairment; however, pre‐dose concentrations were measured at a single time point in this study and may not reflect potential intra‐day variations. Regression analysis of C_max_ and AUC_0–t_ against eGFR as a continuous variable indicated that baseline‐corrected plasma dC and dT exposure increased with decreasing renal function. Although the relationships were significant for C_max_ and AUC_0–t_, correlations were low, suggesting that variables or factors in addition to eGFR may contribute to the large variation in plasma exposures of dC and dT. Moderate‐to‐high variability in the PK profiles of dC and dT has also been observed in previous studies of healthy adult participants in fed and fasted states^26^ and at steady state in participants with TK2d.^28^ This observed variability has been discussed previously.^26^ The clinical relevance of increased dC and dT exposures and their variability in renal impairment remains to be determined.

Regardless of renal function, negligible amounts of intact dC and dT were excreted in urine. The observed increase in the bioavailability metrics of dC and dT in moderate and severe renal impairment is therefore hypothesized to be due to potential decreases in hepatic and extrahepatic metabolism secondary to chronic renal disease. Consistent with results from previous studies (MT‐1621‐103 and MT‐1621‐105),^26^ our findings confirmed that renal excretion of intact dC and dT following a single therapeutically relevant oral dose of doxecitine and doxribtimine in adult participants without renal impairment is a minor excretion pathway. Renal impairment can induce a significant increase in the unbound fraction of highly protein‐bound drugs owing to decreased binding coupled with little or no change in the total clearance (decrease in unbound clearance);^29^, ^30^ however, this is not expected to be a major factor in altered exposures to dC and dT in renal impairment because they are not highly bound (<10%) to human plasma proteins.^23^, ^31^ Published evidence suggests that extensive hepatic and extrahepatic metabolism contribute to the elimination of dC, dT, and their physiological metabolites. Hence, the increase in plasma exposures of dC and dT in renal impairment (and lower CL/F) may be due to decreased hepatic and extrahepatic metabolism and/or increased oral absorption of the nucleosides due to impaired gut barrier function and dysregulation of intestinal nucleoside transporters in chronic kidney disease. Nonrenal clearance by hepatic and extrahepatic drug‐metabolizing enzymes and transporters is known to be affected in chronic kidney disease as a result of increased uremic toxins and/or decreased expression.^32^, ^33^, ^34^, ^35^, ^36^, ^37^ There is no information in the literature on changes in the expression and/or activity of the catabolic enzymes and transporters for dC and dT in chronic kidney disease. Increased renal reabsorption in renal impairment is unlikely to be a meaningful factor because very little dC and dT is excreted intact in urine. Therefore, changes in plasma exposures to dC and dT observed in moderate and severe renal impairment may be due to as‐yet unknown changes in nonrenal clearance mechanisms. Inflammatory factors may be involved in the altered PK of dC and dT in participants with impaired renal function because a systemic inflammatory response is present in chronic kidney disease.^38^ The impact of hepatic impairment on the PK of doxecitine and doxribtimine has not been examined.

In the present study, no safety issues were identified in participants with severe or moderate renal impairment receiving 266.6 mg/kg of doxecitine and doxribtimine (133.3 mg/kg of dC and 133.3 mg/kg of dT). All reported TEAEs occurred in one participant with severe renal impairment; this individual experienced four TEAEs over the course of the study, and these were all considered possibly related to the study drug.

No dose adjustment of doxecitine and doxribtimine is expected for patients with moderate renal impairment based on assessments of safety in the doxecitine and doxribtimine development program, and considering that the drug substances are physiological nucleosides. It is not possible to determine appropriate and effective dose adjustments for this combination drug product in patients with severe or moderate renal impairment because renal impairment has unequal or asymmetric effects on exposure to dC and dT. Doxecitine and doxribtimine is a product in which both drug substances are present in a 1:1 ratio by weight. An appropriate dose adjustment of doxecitine and doxribtimine in patients with renal impairment cannot be determined because renal impairment has distinct effects on both drug substances, and it is not feasible to separately adjust the dosage for doxecitine or doxribtimine contained in the drug product.^12^ Specifying a dose adjustment for doxecitine and doxribtimine solely based on one of the drug substances will result in exaggerated and/or incorrect exposures to the other drug substance, further exacerbating PK variability. The resulting effects of such a dose adjustment on nucleotide pool balance and on mtDNA are unknown, and the clinical implications of these exposure profiles have not been studied in the TK2d patient population. In the absence of any approved therapeutic alternatives, use of doxecitine and doxribtimine in patients with TK2d and renal impairment may be considered at the discretion of the treating physician, accounting for individual patient characteristics, the benefit/risk profile, and severity of the disease. Slower titration may be considered for patients with TK2d with moderate or severe renal impairment.

### Limitations and Generalizability

The study was associated with several limitations. First, our ability to reliably estimate some PK parameters (t_½_, AUC_0–inf_, and dependent parameters) was limited when adequate data were available for only a few participants. In addition, large variations in PK data and the practically determined small sample size, while not unexpected in Phase 1 studies on organ impairment, limited the estimation of exposure changes with greater confidence. For the urine dT concentration data, the fact that the incurred sample reanalysis did not meet acceptance criteria (with no root cause identified after careful investigations) necessitates caution in interpreting results on renal excretion of dT. However, the impact on study results is minimal considering the negligible amount of dT excreted intact in urine irrespective of renal function status, as confirmed in preceding clinical studies. Because there was no matching for concomitant medications at baseline, it was not possible to determine whether any medications for conditions like chronic kidney disease would have affected the PK of dC and dT via acute physiological effects, such as inflammation or changes in blood flow or renal perfusion. However, none of the concomitant medications in either impaired renal function group were known or expected to precipitate PK drug–drug interactions with doxecitine and doxribtimine, and the absorption, distribution, metabolism, and excretion of dC and dT are mediated primarily by non‐CYP enzymes and transporters specific to nucleosides and nucleoside analogs.^12^, ^23^ Systemic or intrarenal inflammation, which may have contributed to the PK profiles of dC and dT in participants with renal impairment, was not measured in this study.^38^ Similarly, although mealtimes and food were standardized throughout the study according to the practices of the clinical research unit where the study was carried out, it is possible that unknown differences in diet may have affected the PK profiles of dC and dT. Finally, metabolites of dC and dT were not analyzed in this study. The metabolism of dC and dT in humans is well understood, and the degradation products are not relevant to the pharmacological activity of doxecitine and doxribtimine in treating patients with TK2d.

## Conclusions

In this study, renal impairment was associated with increased exposure to dC and dT following a single oral administration of doxecitine and doxribtimine. The PK profiles of dC and dT were characterized by high inter‐participant variability, and there was no clear relationship between the degree of renal impairment (moderate/severe) and overall exposure to either component. Additionally, no safety issues were identified in this study of a single oral dose of 266.6 mg/kg of doxecitine and doxribtimine (133.3 mg/kg of dC and 133.3 mg/kg of dT) in participants with severe or moderate renal impairment.

## Author Contributions

All authors made a substantial contribution to study conception, design, execution or analysis, interpretation of data, and drafting or revising the article critically for important intellectual content. All authors have approved the final version of the manuscript.

## Conflicts of Interest

Aravind Mittur is an employee of and stockholder in UCB. Susan VanMeter is an employee of and stockholder in UCB.

## Funding

This study was sponsored by UCB.

## Supporting information



Supplemental Information

## Data Availability

Owing to the small sample size in this study, individual patient‐level data cannot be adequately anonymized because there is a reasonable likelihood that individual participants could be identified. For this reason, data from this study cannot be shared.
