# Supplementary material for: Effect of Renal Impairment on the Pharmacokinetics and Safety of Doxecitine and Doxribtimine: A Single‐Dose Phase 1 Study
Source: Clin Pharmacol Drug Dev. 2026 Feb 13;15(2):e70036. doi: 10.1002/cpdd.70036 (PMC12905029; doi:10.1002/cpdd.70036)
Supplement: Supplementary file 1 — Supplemental Information [file CPDD-15-0-s001.docx]

# Figure S1. Uncorrected plasma concentration–time profiles for (a) dC and (b) dT following a single dose of study treatment (PK population)


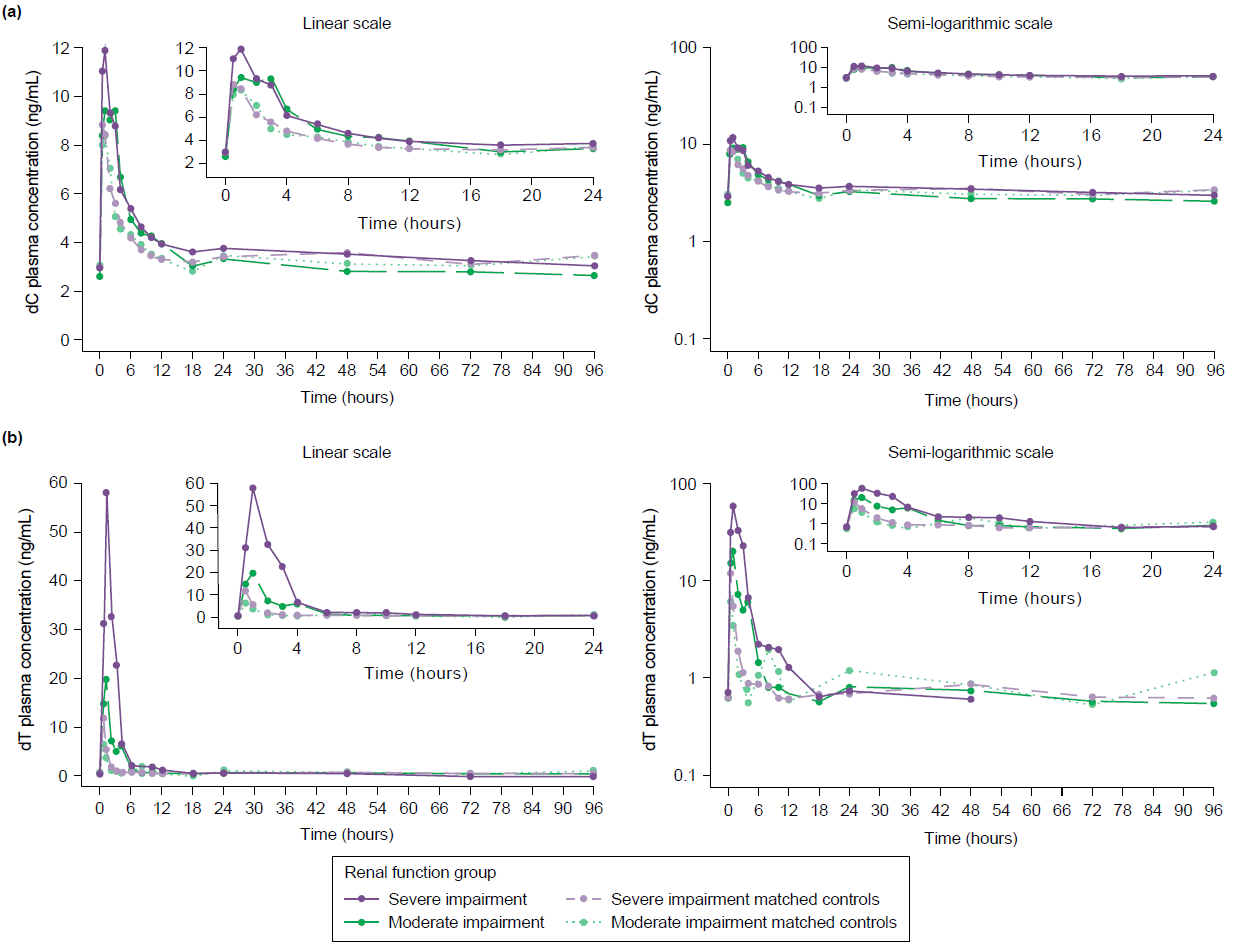


Data shown are mean values. Both linear (left) and semi-logarithmic (right) scales are presented to illustrate concentration changes over time. Lower limit of quantification for both analytes in plasma was 0.500 ng/mL. Part 1 of the study compared participants with severe renal impairment and matched controls. Part 2 of the study compared participants with moderate renal impairment and matched controls.

dC, deoxycytidine; dT, deoxythymidine; PK, pharmacokinetic; SD, standard deviation.

# Figure S2. Uncorrected plasma C_max_ and AUC_0–t_ for (a) dC and (b) dT by renal function group (PK population)


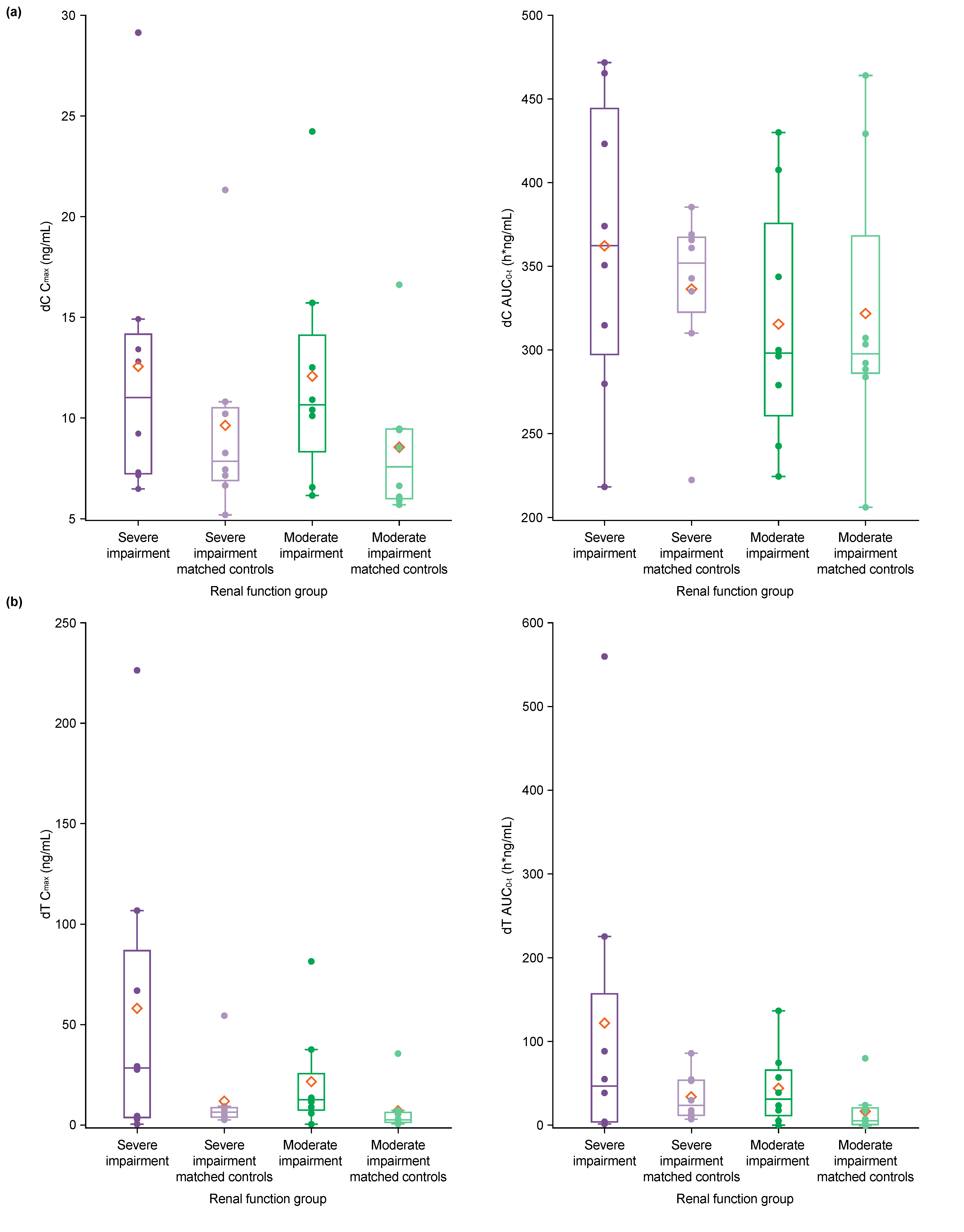


Horizontal lines in the boxes show the median, and top and bottom edges of the boxes show Q3 and Q1, respectively. Diamond symbols show the mean. Lower and upper whiskers in the box plot are the minimum and maximum observed values, respectively, within the range [Q1 − 1.5 × IQR] and [Q3 + 1.5 × IQR], with IQR being the interquartile range (Q3–Q1). Observed values outside this range are marked as outliers (circles above and below the whiskers).

AUC_0–t_, area under the concentration–time curve from time 0 to time of the last quantifiable concentration; C_max_, maximum observed plasma concentration; dC, deoxycytidine; dT, deoxythymidine; IQR, interquartile range; PK, pharmacokinetic; Q, quartile.

# Figure S3. Regression analysis of the relationship between log-transformed baseline-adjusted C_max_ and AUC_0–t_ against renal function based on eGFR. (a) dC exposure; (b) dT exposure (PK population)


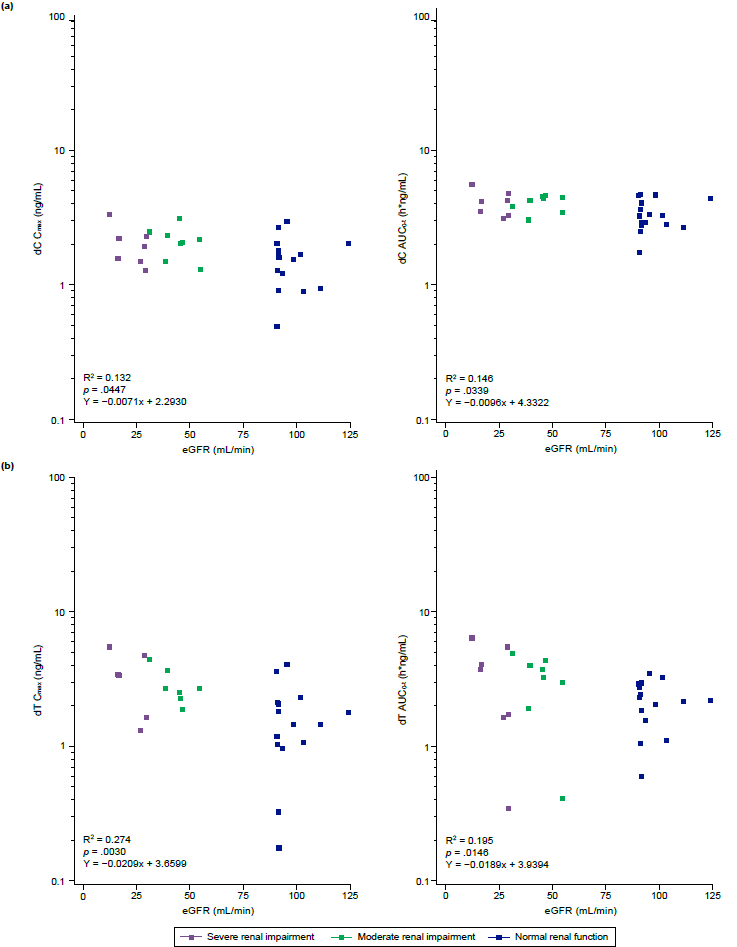


Solid line is the regression line. Shaded area shows 90% CI.
AUC_0–t_, area under the concentration–time curve from time 0 to time of the last quantifiable concentration; CI, confidence interval; C_max_, maximum observed plasma concentration; dC, deoxycytidine; dT, deoxythymidine; eGFR, estimated glomerular filtration rate; PK, pharmacokinetic.

**Table S1.** Effect of Renal Impairment on Baseline-Uncorrected C_max_ and AUC_0–t_ (PK ANOVA population)

| PK Parameter | Renal Function Status | n | Mean (SD) | Geometric LSM | Ratio of Geometric LSM (T/R) | 90% CI for Geometric LSM Ratio (T/R) |
| --- | --- | --- | --- | --- | --- | --- |
| dC | | | | | | |
| C_max,_ ng/mL | Moderate impairment | 8 | 12.1 (5.8) | 11.04 | 1.375 | 0.963, 1.962 |
|  | Matched control | 8 | 8.6 (3.6) | 8.03 |  |  |
|  | Severe impairment | 8 | 12.6 (7.4) | 11.12 | 1.264 | 0.839, 1.905 |
|  | Matched control | 8 | 9.6 (5.1) | 8.79 |  |  |
| AUC_0–t,_ h×ng/mL | Moderate impairment | 8 | 316 (73.4) | 309 | 0.986 | 0.797, 1.219 |
|  | Matched control | 8 | 322 (83.5) | 313 |  |  |
|  | Severe impairment | 8 | 363 (89.5) | 352 | 1.058 | 0.869, 1.289 |
|  | Matched control | 8 | 337 (51.5) | 333 |  |  |
| dT | | | | | | |
| C_max,_ ng/mL | Moderate impairment | 8 | 22.2 (26.4) | 12.26 | 3.021 | 1.061, 8.596 |
|  | Matched control | 8 | 7.7 (11.7) | 4.06 |  |  |
|  | Severe impairment | 8 | 58.5 (76.9) | 20.30 | 2.565 | 0.725, 9.071 |
|  | Matched control | 8 | 12.5 (17.3) | 7.91 |  |  |
| AUC_0–t,_ h×ng/mL | Moderate impairment | 8 | 45.5 (45.0) | 24.2 | 3.373 | 0.914, 12.447 |
|  | Matched control | 8 | 18.1 (26.9) | 7.18 |  |  |
|  | Severe impairment | 8 | 123 (191) | 34.8 | 1.323 | 0.361, 4.847 |
|  | Matched control | 8 | 35.3 (27.9) | 26.3 |  |  |

ANOVA, analysis of variance; AUC0–t, area under the concentration–time curve from time 0 to time of last quantifiable concentration; CI, confidence interval; C_max_, maximum observed concentration; dC, deoxycytidine; dT, deoxythymidine; LSM, least-squares mean; PK, pharmacokinetics; R, reference (matched normal); SD, standard deviation; T, test (severe or moderate impairment).
